# Predictive processes linking sense of agency and fatigue: a novel allostatic framework

**DOI:** 10.3389/fpsyg.2026.1792850

**Published:** 2026-04-02

**Authors:** Emanuela Pizzolla, Angela Marotta, Matthew R. Longo, Mirta Fiorio

**Affiliations:** 1 Department of Neurosciences, Biomedicine and Movement Sciences, University of Verona, Verona, Italy; 2 School of Psychological Sciences, Birkbeck, University of London, London, United Kingdom

**Keywords:** allostasis, fatigue, prediction, sense of agency, sensory attenuation model of fatigue

## Abstract

The sense of agency and the experience of fatigue are usually treated as separate constructs: one concerning perceived control over action, the other reflecting subjective energetic state. However, both rely on how the brain predicts, evaluates, and updates information about the body and its actions. Despite this conceptual proximity, the two phenomena have rarely been examined together, and no unified framework currently explains why changes in perceived control and perceived effort so often co-occur. The aim of this review was to provide such integrative framework. We first examined behavioral, clinical, and neuroimaging evidence indicating that both agency and fatigue rely on the precision of anticipatory models that guide action and bodily regulation. When predictions align with incoming sensory and interoceptive signals, individuals experience a stable sense of control and low perceived effort. When these predictions become imprecise or mismatched, disruptions can arise in both domains. We then evaluated existing theoretical models for agency and fatigue and highlighted the limitations of accounts that treat the two phenomena independently or assume unidirectional relationships. Building on these limitations, we propose an allostasis-based model in which agency and fatigue emerge from the same anticipatory system governing energy regulation. In this view, perturbations in prediction can propagate across systems, producing parallel disruption in perceived control and effort. This new integrative perspective underscores the need for research designs that assess agency and fatigue jointly and provides a conceptual foundation for understanding their co-occurrence across healthy and clinical populations.

## 1 Introduction

The sense of agency and the experience of fatigue are typically treated as belonging to separate domains of human experience: one concerns how individuals perceive themselves as the authors of their actions, the other reflects how costly it feels to sustain those actions (Moore, 2016; Dittner et al., 2004). Despite this conceptual separation, both phenomena display a similar set of characteristics: they are subjective and highly variable, act as regulatory signals that modulate behavior, and are frequently altered across overlapping neurological and psychiatric conditions (Kuppuswamy et al., 2015a; Maurer et al., 2016; Seghezzi et al., 2021). From a phenomenological perspective, the link between agency and fatigue is immediately plausible. On one hand, fatigue not only makes actions physically demanding; it might also alter how actions are perceived. When an activity feels effortful and exhausting, it is often accompanied by a weakened sense of initiating and sustaining it voluntarily. Conversely, a diminished sense of control over one’s actions might be experienced as draining, as if agency required additional effort to be maintained (Ciocan, 2025; Lukitsch, 2020). These experiential overlaps raise a foundational question that has remained largely unaddressed: might the sense of agency and the experience of fatigue depend on shared underlying mechanisms rather than being independent constructs?

To understand this possibility, it is helpful to step back and consider a broader shift in cognitive neuroscience: the growing emphasis on predictive processing as a way to explain perception, action, and interoception (Clark, 2013; Friston, 2010). According to predictive processing frameworks, the brain is fundamentally engaged in anticipating the sensory and bodily consequences of actions and in minimizing the mismatch between these predictions and actual inputs (Friston, 2010; Knill and Pouget, 2004). Subjective experience emerges from this ongoing negotiation between prior expectation and sensory evidence. This predictive framework governs how actions are prepared and monitored, how effort is evaluated, and how physiological needs are anticipated and met.

Seen through this lens, the gap between agency and fatigue becomes less clear-cut. Both phenomena appear intricately tied to how the brain generates, updates, and evaluates predictions about the body. In the agency domain, classic sensorimotor models propose that the feeling of control depends on the match between predicted and actual sensory consequences of movement (Blakemore et al., 1998, 2001). When predictions are precise and accurate, we mark the action as self-generated; when predictions fail, the sense of agency weakens.

In the fatigue domain, predictive models similarly emphasize that fatigue originates from errors in forecasting the cost of action (Kuppuswamy, 2017). Under these accounts, the experience of fatigue not merely reflects metabolic depletion, but emerges from mismatches between predicted effort and the interoceptive evidence received during action.

Yet, despite the conceptual convergence of these phenomena, empirical work directly linking agency and fatigue remains limited. As a result, the field still lacks a unified framework capable of explaining why disturbances in the two domains so often co-occur in both healthy individuals and clinical populations (Kuppuswamy et al., 2015a; Maurer et al., 2016; Seghezzi et al., 2021).

This review aims to provide such a framework by arguing that agency and fatigue are best understood as interconnected expressions of predictive processing. Drawing on behavioral, clinical, and neural evidence, we show how disruptions in predictive mechanisms can simultaneously weaken the sense of control and amplify the experience of fatigue. On this basis, we then introduce an integrative predictive model that extends existing accounts by situating agency and fatigue within a predictive-allostatic framework. This approach not only organizes current findings under a common mechanistic principle but also points toward new avenues for theoretical development, empirical testing, and clinical intervention.

## 2 Agency and fatigue: an overview of distinctive features

### 2.1 Core aspects of agency

The sense of agency can be defined as the subjective experience of being the cause of one’s own actions and their consequences (Haggard, 2017; Moore, 2016). It reflects the feeling of control over one’s movements and the perception of a causal link between an action and its outcome (Grünbaum and Christensen, 2020; Jeannerod, 2003; Lafleur et al., 2020). If these elements are granted, we perceive actions and their consequences as self-produced and distinct from those externally generated. The sense of agency is closely intertwined with other components of bodily self-perception, including body ownership (i.e., the experience of one’s body as belonging to oneself), the underlying processes of which are modulated by individual factors such as age and personality traits (Marotta et al., 2018; Marotta et al., 2016).

Agency is not a unitary phenomenon but unfolds across different levels. An important distinction is between the judgment of agency and the feeling of agency (Synofzik et al., 2008). The judgment of agency denotes a higher-order, explicit, and retrospective evaluation of whether one was the agent of a particular action or outcome. It is more conceptual in nature, influenced not only by sensorimotor information but also by beliefs, contextual cues, and cultural frameworks. The feeling of agency, by contrast, represents an immediate, implicit sense of control that accompanies voluntary action. It arises pre-reflectively, without conscious deliberation, and is thought to rely primarily on predictive sensorimotor processes. Although often aligned, these two levels can diverge, for instance, in clinical populations where bodily sensations of control conflict with explicit judgments about action authorship (Ciaunica et al., 2024; Metcalfe et al., 2012; Moore and Fletcher, 2012).

Building on this distinction, judgment and feeling of agency have been investigated through different approaches (Grünbaum and Christensen, 2020; Moore et al., 2012; Moore, 2016). Judgments are typically assessed with rating scales or questionnaires (Metcalfe and Greene, 2007; Frith, 2013), whereas the feeling of agency is more often examined through implicit phenomena that accompany voluntary action (Synofzik et al., 2008). One example is intentional binding, which refers to the subjective compression of time between a voluntary action and its consequence (Moore and Obhi, 2012). A second well-established phenomenon is sensory attenuation, which refers to the reduced perceived intensity of sensations produced by one’s own actions compared to externally generated ones (Blakemore et al., 2000a; Shergill et al., 2003, 2005). This effect has been consistently demonstrated across different modalities: in audition, self-produced sounds elicit weaker neural responses than external ones (Ford et al., 2007; Kiepe et al., 2021; Mifsud and Whitford, 2017), while in the tactile domain, self-produced touches are perceived as less intense than externally applied touches (Sato, 2008; Weiss et al., 2011).

Further details on the wide range of findings and phenomena associated with the sense of agency can be found in comprehensive reviews of the topic (Haggard, 2017; Moore, 2016). In the present work, “agency” refers to the subjective experience of being the author of one’s actions and their effects (e.g., the feeling that “I am the one causing this movement” when successfully lifting a cup), arising from predictive sensorimotor processes. Accordingly, our account focuses primarily on the pre-reflective and perceptual aspects of agency. It does not address related but distinct constructs, such as self-efficacy (reflective beliefs about one’s abilities; e.g., “I think I can lift the cup”) or autonomy (the degree to which actions are self-endorsed and aligned with one’s values or choices; e.g., “I choose to lift the cup because it aligns with my values”).

### 2.2 Core aspects of fatigue

Fatigue is characterized by substantial terminological variability, and a standardized taxonomy has yet to be established (Skau et al., 2021; Pessiglione et al., 2025). Recent accounts conceptualize fatigue as a multidimensional psychobiological state encompassing experiential, behavioral, and neurophysiological components in different domains (e.g., cognitive or physical) (Pessiglione et al., 2025; Phillips, 2015; Schampheleer et al., 2025). Accordingly, it is typically assessed using a combination of subjective measures (e.g., self-report scales), performance-based indices (e.g., changes in task execution over time), and psychophysiological markers, reflecting its multilevel characterization across psychological, behavioral, and physiological domains (Schampheleer et al., 2025).

Consistent with the aims of this review, to understand how predictive mechanisms shape subjective experience, and particularly how perceived fatigue interacts with perceived control, in the present work we focus specifically on the subjective component of fatigue, defined as an overwhelming sense of tiredness, reduced energy, and perceived exhaustion (Dittner et al., 2004; Kluger et al., 2013; Shen et al., 2006). Subjective fatigue extends beyond a transient “need for rest”, referring to the experience in which sustaining goal-directed activity becomes progressively more demanding from the individual’s perspective (Phillips, 2015). This fatigue dimension is typically assessed through validated self-report instruments designed to capture its qualitative and experiential features (Behrens et al., 2023; Dittner et al., 2004; Enoka and Duchateau, 2008; Kowalski et al., 2021).

The subjective component of fatigue is highly variable, depending on factors such as persistence over time, the domain in which it occurs, and its overall severity. Related to this, subjective fatigue can be described as a temporary state or a trait. State fatigue refers to a transient, situational experience of tiredness in response to acute demands, whereas trait fatigue reflects a more stable and long-term tendency to experience fatigue, usually assessed through retrospective self-report (Enoka et al., 2021; Kowalski et al., 2021). Subjective fatigue can occur across mental and physical domains. On the mental side, it can present as cognitive fatigue, i.e., a feeling of mental tiredness, distractibility, and reduced clarity, often linked to sustained cognitive load (Linnhoff et al., 2019; Pizzolla et al., 2025), or affective fatigue, that is a sense of emotional exhaustion or low motivation, often tied to mood and psychological factors, and frequently reported in depression and addiction (Solomon and Manea, 2022). Physical fatigue, on the other hand, corresponds to the subjective feeling of weakness and loss of energy that follows sustained muscular activity (Billones et al., 2021; Pattyn et al., 2018). In terms of severity, subjective fatigue can range from mild, transient tiredness to a chronic and disabling condition. In healthy individuals, it usually arises in response to temporary demands and is relieved by rest. In contrast, when it becomes persistent, occurs even at rest, and substantially limits everyday functioning, it is considered pathological fatigue (Di Vico et al., 2021; Finsterer and Mahjoub, 2014; Gallagher et al., 2010).

Notably, subjective fatigue is closely related to other dimensions of fatigue, such as performance fatigability and effort, two partially overlapping but conceptually non-equivalent phenomena (Enoka and Duchateau, 2008; Kluger et al., 2013; Tanaka, 2015).

Performance fatigability, refers to the objective decline in motor or cognitive output under sustained demand, e.g., slower reaction times, reduced force, or diminished accuracy (Kluger et al., 2013; Enoka and Duchateau, 2008; Enoka et al., 2021; Tankisi et al., 2023). In the physical domain, fatigability is often associated with neuromuscular fatigue, defined as the progressive loss of a muscle’s ability to produce or maintain required force (Allman and Rice, 2002; Boerio et al., 2005; Edwards, 1981). The relationship between subjective fatigue and performance fatigability remains unclear. Although some accounts propose a substantial overlap between the two (Schampheleer et al., 2025), empirical evidence suggests they are only partially related. Associations between subjective fatigue and objective performance decline are often weak or absent (Andersson et al., 2025; do Espírito Santo et al., 2018), and when present, typically modest in magnitude (Loy et al., 2017). Overall, the available evidence indicates that these two dimensions of fatigue may be related, but they cannot be considered interchangeable and should be assessed independently.

Effort, is widely recognized as a central component in the experience of fatigue, and several definitions explicitly describe subjective fatigue as exhaustion induced by sustained effort (Chaudhuri and Behan, 2004; Matthews et al., 2023). This close relationship becomes clearer when effort is conceptualized not simply as subjective strain (Pageaux, 2016), but as a cost, i.e., the investment of resources such as energy or cognitive control allocated to goal-directed action (André et al., 2026). Within cost–benefit frameworks, actions are sustained when anticipated rewards outweigh perceived costs (André et al., 2026). In this context, subjective fatigue can be understood as emerging when the perceived cost of sustained effort increases or when the expected value of ongoing exertion declines. Empirical evidence supports this view: subjective fatigue tends to increase with prolonged or intense effort, and elevated fatigue is associated with reduced willingness to select high-effort options (Matthews et al., 2023; Müller and Apps, 2019).

Although tightly related, effort and subjective fatigue are not synonymous (De Doncker et al., 2021; de Morree et al., 2012; Kuppuswamy et al., 2015b; Pageaux et al., 2015). Effort reflects the mobilization and perceived cost of resources during action, whereas subjective fatigue reflects the experiential state signaling that this investment has become increasingly taxing or inefficient. Accordingly, individuals may experience effort without subjective fatigue when the investment is perceived as justified or rewarding (Milyavskaya et al., 2021), whereas boredom or low motivation can amplify the perception of fatigue even when effort is minimal (Milyavskaya et al., 2019).

In sum, subjective fatigue describes the *subjective experience* during or after a task, fatigability reflects the body’s or brain’s *objective ability* to perform it, and effort represents the *perceived cost* associated with doing so.

To ensure clarity, the term *fatigue* will be used throughout this review to denote subjective fatigue, unless otherwise specified.

## 3 Current empirical evidence of the link between agency and fatigue

As noted in the introduction, research on agency and fatigue has developed largely in parallel, with few attempts to examine how the two phenomena might interact. Current insights into their link, therefore, come mainly from indirect evidence, particularly from studies that manipulate effort to test its influence on agency. This approach, however, has produced mixed and sometimes conflicting results (Hon et al., 2013; Van den Bussche et al., 2020; Lafargue and Franck, 2009).

On one hand, some studies indicate that increased effort can strengthen agency, especially when action–outcome contingencies are ambiguous or uncertain. For instance, Demanet et al. (2013) reported that greater physical effort, even when unrelated to the task (e.g., pulling a resistance band during an intentional binding task), increased the feeling of agency. Similarly, Minohara et al. (2016) reported stronger explicit agency ratings when actions required greater intentional effort under temporal uncertainty. These results suggest that effort may act as an additional predictive cue reinforcing the experience of control when external information is unreliable.

On the other hand, other studies have reported the opposite pattern: increased effort can diminish the sense of agency (Vastano et al., 2017). Hon et al. (2013) observed that higher cognitive load weakened explicit agency measures, while Howard et al. (2016) demonstrated that greater perceived task effort reduced intentional binding.

A useful attempt to reconcile these conflicting results comes from Van den Bussche et al. (2020), who proposed that the *predictability* of effort may play a key role. When effort is anticipated, i.e., when the task is expected to be demanding, the exertion is interpreted as part of a planned, voluntary action, thereby enhancing agency (Demanet et al., 2013; Minohara et al., 2016). However, when the effort required is unexpected, unwanted, or disproportionate to what was predicted, this creates a prediction error between anticipated and actual task demands. In these cases, the mismatch can undermine agency and produce a subjective feeling of reduced control (Hon et al., 2013; Howard et al., 2016).

The mixed pattern observed in healthy participants also highlights a broader issue: if transient effort can either enhance or weaken agency depending on how it is predicted, then disorders marked by chronic effort dysregulation may show even more pronounced alterations in the feeling of control. Clinical populations in which pathological fatigue is a prominent symptom may therefore provide a natural context to examine whether these effort–agency dynamics extend beyond laboratory manipulations.

A first line of evidence comes from functional movement disorders (FMD), a condition characterized by motor symptoms that outwardly resemble voluntary movements (e.g., functional tremor may momentarily disappear with distraction) but are experienced by patients as involuntary (Edwards et al., 2013; Fiorio et al., 2022). This dissociation, where seemingly intentional actions are not perceived as self-generated, points to a disruption in the sense of agency. Consistent with this view, several studies have reported marked alterations in implicit measures of agency in FMD. Specifically, patients exhibit reduced sensory attenuation both the behavioral level (Pareés et al., 2014) and neurophysiological level, as indicated by the absence of the typical movement-related suppression of somatosensory evoked potentials (Macerollo et al., 2015). Explicit agency judgments, by contrast, tend to remain relatively preserved (Marotta et al., 2017), pointing to a dissociation between explicit and implicit components of agency in FMD patients. Crucially, however, none of these studies categorized patients based on pathological fatigue, one of the most disabling and prevalent non-motor symptoms of FMD, affecting over 75% of patients (Di Vico et al., 2021). Given the high prevalence and clinical impact of fatigue in FMD, it is plausible that at least part of the reported agency alterations may be attributable to fatigue itself, rather than reflecting FMD pathology alone. However, to date, no study has systematically investigated the relationship between fatigue and alterations in the sense of agency in FMD, leaving this potential interaction largely unexplored.

Another informative case is Parkinson’s disease (PD), in which fatigue is among the most common and disabling non-motor symptoms, affecting up to half of patients and often proving resistant to treatment (Di Vico et al., 2021; Siciliano et al., 2018). In parallel with these high fatigue levels, several studies have examined alterations in predictive processes relevant to agency (Saito et al., 2017; Moore et al., 2010a; Wolpe et al., 2018). Across different paradigms, agency in PD is not uniformly disrupted but appears highly sensitive to dopaminergic modulation. For example, implicit agency, measured through intentional binding, is comparable to controls when patients are off medication, yet increases with dopaminergic treatment (Moore et al., 2010a). Sensory attenuation exhibits a similar pattern: although group-level differences from controls are minimal, within patients, it decreases with increasing motor severity and increases with higher dopaminergic dose (Wolpe et al., 2018). These findings suggest that predictive precision is progressively compromised as the disease advances, while dopaminergic therapy can partly restore it. Importantly, fatigue was not assessed in these studies; however, its high prevalence in PD leaves open the possibility that some variability in agency-related measures may be influenced by fatigue symptoms themselves rather than by disease severity alone.

A further line of evidence comes from schizophrenia and major depressive disorder (MDD), two psychiatric conditions in which both fatigue and agency disturbances are frequent and disabling. In schizophrenia, fatigue affects a substantial proportion of patients and is closely linked to poorer functional health and reduced quality of life (Waters et al., 2013; Laraki et al., 2023). Converging evidence also shows marked disruptions in agency, including reduced sensory attenuation in both auditory and somatosensory domains (Blakemore et al., 2000b; Shergill et al., 2005; Rossetti et al., 2024) and abnormal intentional binding, indicating difficulties in predicting the temporal relation between actions and their outcomes (Franck et al., 2005; Voss et al., 2010; Graham-Schmidt et al., 2016; Roth et al., 2023).

Comparable patterns emerge in MDD, where patients frequently exhibit heightened levels of fatigue (Ratcliffe, 2015), as evidenced by diminished voluntary actions and increased perception of effort, even after mood symptoms remit (Cléry-Melin et al., 2011). Here too, recent studies demonstrate reduced intentional binding and diminished explicit agency judgments, with impairments scaling with symptom severity (Scott et al., 2022; Vogel et al., 2024). A potential bridge between these agency disturbances and the high prevalence of fatigue is provided by Lin et al. (2024), who demonstrated that effort becomes a critical determinant of agency in MDD. The sense of control was strengthened when effortful actions led to success but shifted into a maladaptive pattern (marked by self-blame) when the expected outcome was not achieved.

Additional indications come from Gilles de la Tourette syndrome (GTS) and stroke. In GTS, fatigue is not a core diagnostic feature, but it is nonetheless frequently reported, often in relation to tic exacerbation, and contributes significantly to reduced quality of life (Conelea and Woods, 2008; Cohen et al., 2013). Agency has also been examined in this condition, with intentional binding studies revealing significantly reduced binding (Moccia et al., 2024; Zapparoli et al., 2020a). In stroke, fatigue is among the most common and disabling long-term consequences (Kuppuswamy, 2022). Similarly, patients have been found to display altered agency and misattribute action outcomes (Miyawaki et al., 2020, 2022).

Although current evidence connects effort and agency more directly than fatigue and agency, these findings highlight a shared reliance on predictive processes surrounding effort estimation. Effort is a key variable in both domains: its predictability shapes agency, and its misestimation is central to the development of fatigue. Agency and fatigue, therefore, are best understood not as separate domains, but as interconnected expressions of predictive and perceptual processes.

## 4 Agency and fatigue: shared mechanisms and brain networks

### 4.1 Predictive models of agency and fatigue

The evidence reviewed so far suggests that both agency and fatigue are shaped by the fit between what is expected and what is experienced, meaning that both constructs are inherently predictive, relying on the brain’s ability to anticipate action outcomes, internal states, and task demands (Kuppuswamy, 2017; Marshall et al., 2018; Sato and Yasuda, 2005). This raises the question of whether existing theoretical models capture this predictive structure. Two models are especially relevant in this regard: the Comparator Model, which has long been considered a central account of how the sense of agency arises, and the Central Governor Model, a leading framework for understanding the regulation of fatigue.

The Comparator Model remains the canonical predictive account of the sense of agency (Blakemore et al., 1998, 2001). When a voluntary action is initiated, an efference copy of the motor command is fed to a forward model to predict the imminent sensory consequences of that action. If the prediction matches the actual feedback, the resulting sensation feels less salient (sensory attenuation), helping to mark the action as self-produced and reinforcing the experience of agency, i.e., the pre-reflective feeling that “I am the one causing this” (David et al., 2008; Haggard, 2017). Conversely, when prediction and feedback diverge, prediction error grows, attenuation is reduced, and the sense of control weakens. The model, therefore, explains why implicit markers such as intentional binding and sensory attenuation tend to track the feeling of agency in controlled tasks: both reflect the success of predictions about one’s own action consequences. Although subsequent work has expanded agency theory, incorporating the weighting of external cues (Moore and Haggard, 2008; Moore and Fletcher, 2012; Synofzik et al., 2013), hierarchical distinctions between levels of attribution (Pacherie, 2008), and retrospective inferential processes (Wegner and Wheatley, 1999; Wegner, 2002; Lafleur et al., 2020), the Comparator framework remains the core sensorimotor formulation of how predictive signals contribute to action authorship and control, and why mismatches between expected and actual outcomes reduce them (Moore and Haggard, 2008; Moore and Fletcher, 2012).

An analogous predictive logic organizes the Central Governor Model of fatigue (St Clair Gibson and Noakes, 2004). The model conceptualizes fatigue as a protective mechanism, whereby a central control system integrates sensory signals from different systems (cardiovascular, thermoregulatory, metabolic) to prevent potential harm. When the brain predicts that further exertion would compromise physiological integrity, it generates the experience of fatigue to limit performance (Abbiss and Laursen, 2005; Pattyn et al., 2018; Tornero-Aguilera et al., 2022). The controller, therefore, modulates motor output through feedforward adjustments; when predicted costs climb relative to available reserves or acceptable risk, the subjective experience of fatigue increases and behavior adapts (slowing, stopping, or seeking rest). In this model, fatigue is experienced as the result of altered homeostasis (Gibson et al., 2003; Pattyn et al., 2018) and can be more generally described as a mental representation of the physiological changes occurring in the body (Damasio, 1999; Gibson et al., 2003). Critiques of the model have targeted its limited consideration of more reflective elements, such as motivation, emotions, and goals (see the Psychological-Motivational Model by Marcora, 2008, and the Motivational Intensity Theory by Wright, 1996, for details). However, even these debates take for granted the Central Government’s main claim: fatigue is not a simple readout of spent resources but a forecast about the future costs of continuing to act.

Set side by side, the two models converge on several points that are central to this review. Both treat experience as prediction-dependent: agency hinges on forecasts of sensory consequences; fatigue hinges on forecasts of energetic costs. Both rely on comparison operations that register the fit (or misfit) between expected and actual states, whether those states are exteroceptive/proprioceptive outcomes of movement or interoceptive signatures of bodily demand. Finally, both imply adaptive adjustment: when mismatches grow, the system re-weights signals and changes behavior by reducing authorship attributions in agency tasks or by throttling effort and seeking recovery under increasing cost.

These commonalities do not erase important differences. The Comparator Model focuses on fine-grained, millisecond-scale predictions about the sensory consequences of discrete actions, whereas the Central Governor emphasizes slower, integrative predictions about sustaining action under physiological constraints. Yet taken together, they support the same thesis: agency and fatigue are perceptual outcomes of anticipatory computations that compare what the organism expects to happen with what actually unfolds.

### 4.2 Shared brain networks supporting predictive mechanisms

The predictive models outlined above suggest that both agency and fatigue rely on the brain’s capacity to generate expectations, compare them with incoming signals, and adjust behavior when mismatches occur. This theoretical convergence motivates a closer look at the neural level to determine whether partially overlapping brain systems may contribute to both phenomena. While no study has yet demonstrated a common neural basis directly linking agency and fatigue, neuroimaging work in each domain highlights several candidate regions that support prediction, multisensory integration, and error monitoring (Sperduti et al., 2011). In particular, the insula, the supplementary motor area (SMA and pre-SMA), the prefrontal cortex, and the cerebellum have each been implicated in research on agency and on fatigue (Hogan et al., 2020; Seghezzi et al., 2019a, 2019b). Although their roles are not identical, and evidence remains indirect, these hubs can be considered as part of a distributed predictive control system potentially relevant to both constructs.

#### 4.2.1 The insula

The insula plays a crucial role in interoceptive processes (Critchley et al., 2004; Schulz, 2016), encoding information from homeostatic shifts and internal body signals (Craig, 2009; Hanken et al., 2014; Liu et al., 2018). In the domain of agency, the insula contributes to comparing motor prediction with the actual outcomes of voluntary actions (Sperduti et al., 2011; Tisserand et al., 2023). This is evident in studies linking insular activation to self-attribution of movement (Farrer and Frith, 2002) and to the sense of body ownership in paradigms like the Rubber Hand Illusion (i.e., a protocol that induces an illusory ownership over a fake hand) (Tsakiris et al., 2007). Disruptions in insular function can impair motor prediction and alter the sense of control over actions (Farrer and Frith, 2002; Nahab et al., 2011), as seen in anosognosia for hemiplegia, where patients mistakenly believe they retain control over paralyzed limbs (Karnath and Baier, 2010; Sperduti et al., 2011), and in schizophrenia, where insular abnormalities are associated with misattributions of agency and self-monitoring deficits (Maeda et al., 2013; Sheffield et al., 2020; Kozáková et al., 2020).

The insula is also crucially involved in the perception of effort and perceived fatigue, consistent with its role in processing sensory signals related to shifts in internal homeostasis (Kuppuswamy, 2022). Clinical evidence underscores this contribution across several conditions. In PD and multiple sclerosis, increased fatigue has been associated with reduced insular metabolism, cortical thinning, and altered connectivity with prefrontal and somatosensory areas (Andreasen et al., 2010; Cho et al., 2017; Filippi et al., 2002; Hanken et al., 2014). In MDD, increases in subjective vitality, a proxy for reduced fatigue, have been associated with strengthened connectivity between the posterior insula and sensorimotor areas (Xu et al., 2020), echoing broader findings of insular abnormalities and atypical interoceptive processing in this disorder (Fitzgerald et al., 2008; Mayberg, 2003; Liu et al., 2010; Wiebking et al., 2010). Such alterations are thought to heighten the salience of internal bodily signals (Garcia-Cebrian et al., 2006; Nyboe Jacobsen et al., 2006), providing a plausible neural basis for the increased perception of effort frequently reported in this clinical group.

Given the insula’s central role in predictive processing and monitoring internal bodily states, it is well-positioned to mediate the interaction between agency and fatigue. In both constructs, the insula may function as a comparator, detecting mismatches between predicted and actual outcomes. For the agency, this involves discrepancies between intended actions and their sensory consequences; for fatigue, it may involve mismatches between expected internal change and actual shift in homeostasis.

#### 4.2.2 Supplementary motor area

The Supplementary Motor Area (SMA) and pre-SMA are central hubs for motor planning, action selection, and voluntary control (Nachev et al., 2007; Sperduti et al., 2011). The anterior pre-SMA, in particular, plays a key role in action selection and motor inhibition (Cunnington et al., 2003; Kennerley et al., 2004; Krieghoff et al., 2009). Through their involvement in selecting, initiating, monitoring, and suppressing actions, these regions are positioned to make critical contributions to both the sense of agency and the perception of effort.

In the context of agency, numerous studies have linked SMA and pre-SMA activity to implicit measures, such as temporal binding (Cavazzana et al., 2015; Zapparoli et al., 2020b). Disrupting pre-SMA excitability through repetitive transcranial magnetic stimulation (i.e., theta-burst stimulation protocol; Moore et al., 2010b) or transcranial direct current stimulation (Cavazzana et al., 2015) significantly alters temporal binding, demonstrating its causal contribution to agency. More broadly, pre-SMA is regarded as a key node for resolving intention-feedback discrepancies and for monitoring whether actions unfold as predicted (Sperduti et al., 2011). Clinical evidence converges with these experimental findings: FMD patients, who often experience a diminished sense of voluntariness on their motor symptoms (Delorme et al., 2016), show selective dysfunction in SMA and pre-SMA regions (Maurer et al., 2016; Nahab et al., 2017), and individuals with Tourette syndrome exhibit altered activity in these regions, consistent with impaired action monitoring in the presence of semi-voluntary tics (Zapparoli et al., 2020a). These patterns align with proposals that effective connectivity between pre-SMA and parietal regions is a core neural substrate supporting the sense of agency (Haggard, 2017).

SMA and pre-SMA also contribute to the experience of fatigue. Inhibiting SMA activity through repetitive transcranial magnetic stimulation has been shown to reduce perceived physical effort (Emanuel et al., 2021; Zénon et al., 2015), suggesting a role in encoding signals of physical exertion. Consistent with this interpretation, altered activation and connectivity within SMA and pre-SMA have been associated with pathological fatigue in clinical populations (Siciliano et al., 2020; Tessitore et al., 2016). In drug-naïve patients with early Parkinson’s disease, fatigue severity correlates with increased pre-SMA–parietal connectivity and decreased connectivity between the SMA and the left middle frontal gyrus (Siciliano et al., 2020). This pattern points to a disruption within fronto-parietal predictive networks that are also implicated in action monitoring and agency (Haggard, 2017). One possible interpretation is that altered connectivity reflects inefficient attenuation of proprioceptive or somatosensory signals ascending to higher-order motor regions (Siciliano et al., 2020).

Together, these findings support the view that the SMA and pre-SMA contribute to both the sense of agency and the regulation of fatigue. Disruptions within this network may therefore help explain why altered movement control frequently co-occurs with heightened subjective fatigue and a weakened sense of agency.

#### 4.2.3 Prefrontal cortex

Formulating intentionality to act is a crucial prerequisite to distinguishing between voluntary and involuntary actions (Vinding et al., 2013). The intentional process engages specific brain regions associated with action selection and movement planning. In this regard, the prefrontal cortex (PFC) plays a prominent role (Ridderinkhof et al., 2004).

Within this network, the dorsolateral prefrontal cortex (DLPFC) has been repeatedly implicated in the sense of agency, particularly in contexts requiring the generation and selection of competing action alternatives (Haggard, 2017). Experimental modulation of the DLPFC through transcranial direct current stimulation has proved to enhance intentional binding in situations involving the selection between alternative actions, suggesting a causal contribution to agency-related computations (Khalighinejad et al., 2016). Beyond the DLPFC, other prefrontal regions, such as the medial PFC (mPFC), also contribute to self-attribution processes. Active modulation of mPFC using transcranial magnetic stimulation improved self-other discrimination in a reality monitoring task (Subramaniam et al., 2020), a pattern mirrored in schizophrenia, where reduced mPFC engagement is linked to impairments in self-agency and reality monitoring (Subramaniam, 2021). The PFC’s contribution to agency also depends on its coordinated interactions with parietal regions, such as the angular gyrus, which is responsible for detecting discrepancies between predicted and actual sensory feedback (Farrer and Frith, 2002).

Beyond its role in agency, the PFC is also central to the experience of fatigue. The basal ganglia-thalamus-PFC circuit is considered one of the crucial networks involved in the pathophysiology of fatigue in clinical conditions (Capone et al., 2020; Chaudhuri and Behan, 2004; Wylie et al., 2020). Disruptions in the non-motor functions of the basal ganglia and their connections with the thalamus and PFC are thought to interfere with the integration of sensory, motor, and affective signals, thereby amplifying perceived effort and, consequently, contributing to fatigue arising (Chaudhuri and Behan, 2004; Dobryakova et al., 2013; Kuppuswamy, 2022). Structural and metabolic studies corroborate this view: reduced glucose metabolism within this network is associated with fatigue severity in multiple sclerosis (Roelcke et al., 1997), and reduced perfusion in the thalamus and basal ganglia similarly tracks higher fatigue levels (Inglese et al., 2007). Lesion studies provide further support, showing that damage to ventromedial prefrontal regions disproportionately increases fatigue relative to other prefrontal lesions (Pardini et al., 2010). Additional evidence comes from neurochemical studies, which highlight alterations in dopaminergic and serotonergic pathways within the BG-PFC loop in fatigued patients (Pavese et al., 2010; Yamamoto et al., 2004).

The emerging picture positions the PFC as a core node in the predictive processes underlying agency and fatigue, influencing both authorship judgments and the perception of effort.

#### 4.2.4 Cerebellum

Although traditionally associated with motor coordination and control (Bastian, 2006; Manto et al., 2012), the cerebellum also plays an important role in non-motor domains such as self-motion perception (Baumann et al., 2015), action-feedback comparative process (Blakemore et al., 1998; Tanaka et al., 2020), homeostatic regulation (Casamento-Moran et al., 2023), and bodily self-perception (Fiorio et al., 2014; Marotta et al., 2021). Its function in predictive mechanisms and error correction makes it a key region for both the sense of agency and the perception of fatigue.

In the context of agency, the cerebellum is thought to contribute to the comparison between predicted and actual sensory consequences of action (Blakemore et al., 2001; Marotta et al., 2021; Welniarz et al., 2021). Early studies demonstrated increased activation in the right cerebellar cortex when expected tactile consequences of self-generated movement were absent (Blakemore et al., 1998), and when larger temporal discrepancies separated actions from their effects (Blakemore et al., 2001; Kilteni et al., 2023). More recent work shows that cerebellar activity correlates with implicit markers of agency such as temporal binding (Zapparoli et al., 2020b), and with sensitivity to action–outcome delays during self-initiated movement (van Kemenade et al., 2019). Clinical findings further support this role: cerebellar dysfunction is frequently observed in conditions marked by disrupted motor awareness and impaired voluntary control, including Tourette syndrome and dystonia (Neychev et al., 2008; Welniarz et al., 2021; Zapparoli et al., 2020a).

The cerebellum’s relevance extends to fatigue as well, particularly in relation to movement control and interoceptive regulation. Experimental evidence shows that reduced cerebellar activity following fatigue-inducing tasks is associated with increased movement variability, lower physical fatigue, and impaired motor control (Casamento-Moran et al., 2023). In clinical populations, such as patients with spinocerebellar ataxia, cerebellar damage has been associated with difficulties in managing bodily homeostasis and increased reports of fatigue (Casamento-Moran et al., 2023; Capone et al., 2020; Novo et al., 2018). These patterns suggest that the cerebellum contributes to interoceptive calibration, influencing how precisely and consciously internal signals related to fatigue are perceived.

Given its central role in integrating sensorimotor predictions with interoceptive feedback, the cerebellum can be viewed as a bridge region linking the sense of agency with the experience of fatigue.

#### 4.2.5 Anterior cingulate cortex

The anterior cingulate cortex (ACC), particularly its dorsal portion (dACC), is a key hub for performance monitoring and control, integrating information about effort, reward, and internal state (Kuppuswamy, 2022). It has long been implicated in detecting errors and monitoring conflict, with activity scaling as actions deviate from intended goals or expected outcomes (Carter and van Veen, 2007). Situated within the broader cortical midline structures (CMS), the dACC forms part of a functionally integrated medial system encompassing ventromedial and dorsomedial prefrontal cortices as well as posterior cingulate regions (Northoff et al., 2006). Meta-analytic evidence indicates that cortical midline structures are consistently engaged across diverse forms of self-referential processing, independent of domain, suggesting a supramodal role in integrating information relative to the self (Northoff et al., 2006).

This contribution is particularly relevant in the context of agency, where CMS are consistently engaged during action monitoring and self-generated behavior (Crivelli and Balconi, 2017; Seghezzi et al., 2019a). More specifically, the ACC contributes to evaluating whether ongoing actions match higher-order intentions and task rules, supporting adjustments in control when discrepancies arise (Seghezzi et al., 2019a). Clinical studies further demonstrate that altered cingulate activation is associated with disturbances of the sense of agency, particularly in schizophrenia, where hypoactivation within CMS accompanies impaired self-referential processing. Spaniel et al. (2016) reported reduced activation in the left medial frontal gyrus and posterior cingulate cortex during self-agency judgments in first-episode schizophrenia patients relative to healthy controls. These findings support the view that agency relies on predictive monitoring mechanisms embedded within medial frontal circuitry. Given its established role in error detection and outcome monitoring, the dACC is well positioned to signal mismatches between intended and observed consequences of action, thereby contributing to the maintenance of authorship.

Converging evidence also implicates the dACC in fatigue and effort-based regulation. Functional neuroimaging studies consistently identify the dACC as a core node within a “fatigue network,” alongside dorsolateral prefrontal cortex, ventromedial prefrontal cortex, striatum, and anterior insula (Wylie et al., 2020). Connectivity analyses reveal that increasing cognitive fatigue is associated with decreased connectivity between these regions, suggesting network-level reorganization rather than isolated regional dysfunction (Wylie et al., 2020). Task-based studies show that dACC activation decreases as mental fatigue emerges and performance declines, and that reward incentives can restore both behavioral performance and dACC engagement (Darnai et al., 2023). Clinical findings reinforce this association. In multiple sclerosis, fatigue severity has been linked to reduced gray matter volume in right ACC and midcingulate regions (Gonzalez Campo et al., 2020) and altered cingulate recruitment patterns during task performance, consistent with compensatory control mechanisms (Tartaglia et al., 2008). Similarly, smaller baseline dorsal ACC gray matter volume has been associated with higher fatigue in large samples of individuals following mild COVID-19 infection (Niu et al., 2025).

Together, these findings position the ACC as a comparator and valuator that, in both agency and fatigue, detects mismatches between intended and actual states and updates the perceived worth of continued control, thereby shaping both the sense of agency and the experience of fatigue.

## 5 Integrating agency and fatigue within a predictive framework: from the sensory attenuation model of fatigue to a new Allostasis-based perspective

### 5.1 The sensory attenuation model of fatigue

The behavioral, clinical, and neural evidence reviewed so far highlights several points of convergence between the sense of agency and fatigue; yet, until recently, no theoretical account has explicitly sought to integrate them. The Sensory Attenuation Model of Fatigue (SAF model), proposed by Kuppuswamy (2017), represents a first attempt to do so by linking predictive mechanisms, effort perception, and agency disruption, particularly in the context of pathological fatigue.

The SAF builds on the well-established phenomenon of sensory attenuation, i.e., the reduction in perceived intensity of self-generated sensations compared to externally produced ones (Shergill et al., 2003; Dewey and Knoblich, 2014; Hughes et al., 2013). As outlined earlier in this review, sensory attenuation has been widely considered an implicit marker of the sense of agency because it reflects the brain’s ability to predict and down-regulate the sensory consequences of its own actions, reinforcing authorship. The SAF model extends this principle to fatigue. When the sensory attenuation function is normal, predictions about action outcomes, including the required effort, are accurate, and afferent signals from self-generated movement are appropriately suppressed. Under these conditions, actions feel relatively effortless, agency is preserved, and fatigue remains low. When sensory attenuation is reduced or absent, however, afferent signals are insufficiently suppressed, and the predicted ease of movement no longer matches incoming sensory evidence. This mismatch manifests as increased perceived effort, giving rise to the subjective experience of fatigue (Kuppuswamy, 2017, 2022).

The novelty of the SAF model lies in its reconceptualization of fatigue as a perceptual state grounded in altered effort perception. Rather than being a simple consequence of peripheral exhaustion or resource depletion, fatigue is framed as an abnormal inference about the cost of action. The SAF model was directly inspired by clinical observations: patients with pathological fatigue, such as those with stroke, multiple sclerosis, or PD, frequently report limb heaviness and marked effortfulness despite preserved motor strength (Kuppuswamy, 2017, 2022). These dissociations suggest that pathological fatigue does not stem from muscle output but from a distorted perception of effort, arising when afferent input from muscle tone is not adequately suppressed.

Despite its value, the SAF model also presents important limitations. It primarily addresses pathological fatigue and offers limited explanation for transient, physiological forms of fatigue. Moreover, it does not fully account for the mixed findings on how effort influences agency in healthy participants (Demanet et al., 2013; Hon et al., 2013; Howard et al., 2016). Nevertheless, by directly connecting effort perception and agency disruption, the SAF model provides an important foundation for developing more comprehensive and integrative accounts of agency and fatigue.

### 5.2 Toward an extended allostasis-based predictive model of agency and fatigue

As anticipated, the SAF model (Kuppuswamy, 2017) provides an important first step toward linking agency, effort, and pathological fatigue within a predictive framework. However, its scope is necessarily narrow: by focusing primarily on sensory attenuation, it captures one mechanism through which effort perception may become distorted, but it does not fully account for the diversity of fatigue experiences across dimensions, nor for the bidirectional influences between fatigue and agency observed in both healthy and clinical populations. To move beyond these constraints, a broader predictive framework is needed.

The concept of allostasis offers such a framework. Allostasis refers to the principles that regulate the body’s resource use through anticipatory control (Jungilligens et al., 2022). It involves active predictive mechanisms by which the nervous system estimates future needs and adjusts ongoing demands using both interoceptive signals and contextual information (Jungilligens et al., 2022). In contrast to homeostasis, which aims to re-establish a pre-existing physiological condition, allostasis seeks to achieve metabolic efficiency by ensuring that resources are allocated according to the body’s specific needs *before* those needs are experienced.

Allostatic theories describe energy resources and cognitive control as limited and costly resources that must be dynamically allocated based on expected benefits and anticipated costs (André et al., 2026). Within such cost–benefit frameworks, the nervous system estimates the expected value of exerting effort (i.e., the cost of actions), integrating reward prospects, predicted energetic and cognitive control demands, and internal state variables. From this perspective, energy supply and cognitive control expenditure become part of the organism’s allostatic budget: deploying control consumes metabolic and neural resources, and its allocation is regulated to prevent inefficiency or overload.

**Figure 1:**
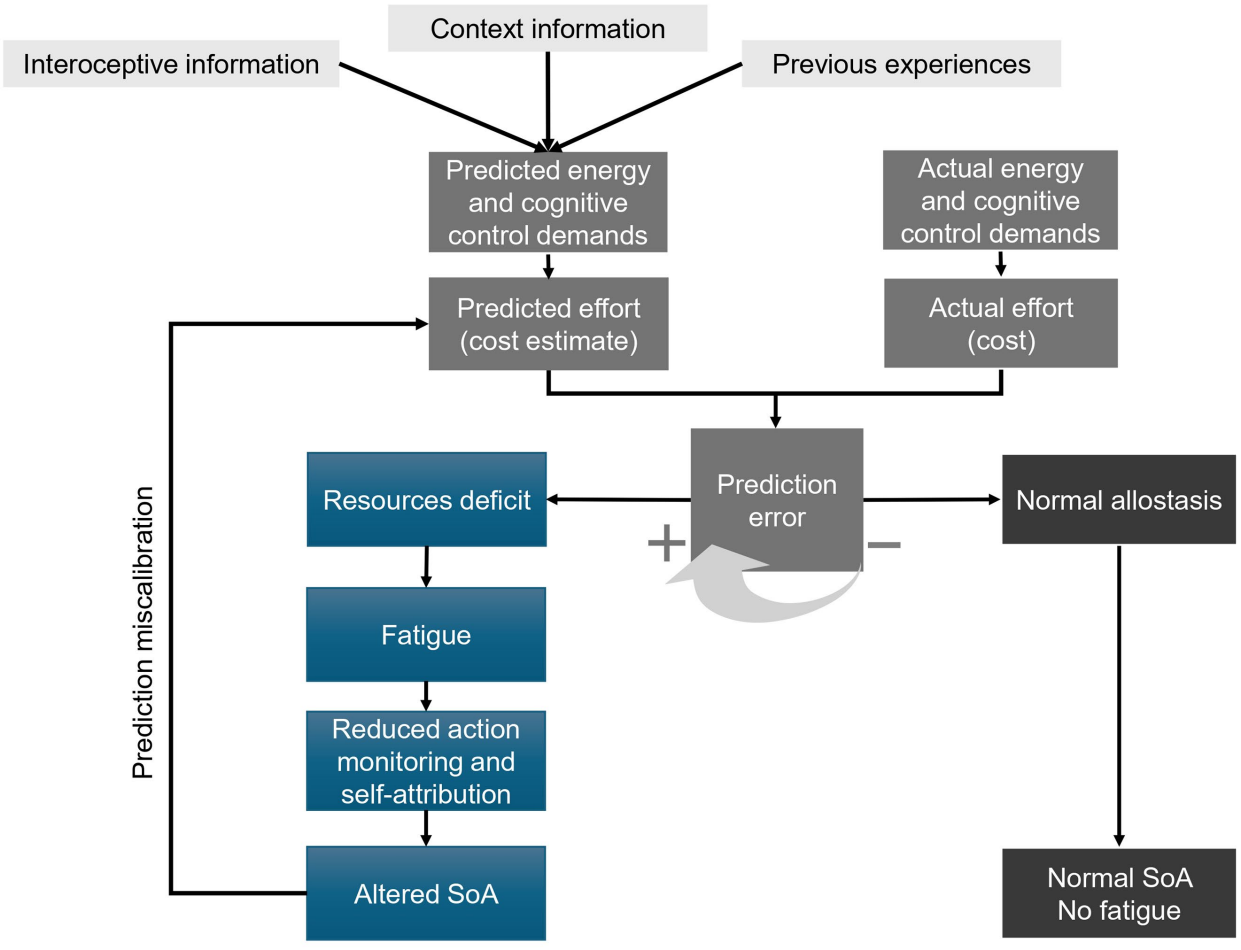
Allostasis-based predictive model linking fatigue and sense of agency. Top to bottom: *Interoceptive signals*, *contextual information*, and *previous experiences* are integrated to generate *predictions about energetic and control demands* required for an action. These predictions inform the estimate of *predicted effort* (anticipated action cost). In parallel, the system registers the *actual energetic and control demands* associated with the task and the *actual effort* required to perform it. The comparison between predicted effort and actual effort generates a *prediction error*, reflecting the discrepancy between expected and experienced costs. When prediction error is minimized (*−*), the system maintains *normal allostatic regulation*, allowing appropriate resource allocation and resulting in a *normal sense of agency (SoA) without fatigue*. However, when prediction errors increase (+), the system infers a *resource deficit*, leading to the subjective experience of *fatigue*. Fatigue reduces the resources available for *action monitoring and self-attribution*, producing an *altered sense of agency*. Crucially, the model also proposes a recursive feedback loop. When the sense of agency is altered, the accuracy of predictions about action outcomes and their energetic costs may deteriorate. This *prediction miscalibration* feeds back into the estimation of *predicted effort*, increasing the likelihood of future mismatches between predicted and actual costs. In this way, fatigue and altered agency can mutually reinforce each other through ongoing prediction errors within the allostatic control system.

This broader predictive principle enables the co-location of effort, fatigue, and agency within the same predictive system. Effort can be as predictive signal that informs goal-directed systems about the anticipated energy and control required to achieve an outcome. Fatigue, in contrast, serves as an adaptive signal, indicating a temporary resource shortfall and prompting behavioral changes to preserve metabolic and cognitive efficiency (Di Vico et al., 2021; Raizen et al., 2023). When energetic or control availability is reduced, fewer resources are available for the cognitive and motor operations that support agency, such as predicting sensory outcomes or monitoring discrepancies. As a result, the sense of control may be weakened (Baumeister et al., 2007; Evans et al., 2016). From this perspective, fatigue functions as a cog within the organism’s allostatic machinery, limiting the self-attribution processes and enabling faster recovery of energy. Agency alterations experienced during fatigue can therefore be seen not simply as failures of action monitoring but as a form of compensation exerted by the body to predict the upcoming energy and cognitive needs and maintain allostatic balance (Figure 1).

The relationship, however, is not unidirectional. When the sense of agency is compromised, predictions about the consequences of actions, including the effort they require, become less accurate. This miscalibration of costs can lead to overestimation of energetic and control needs, amplifying the subjective experience of fatigue. In this view, agency and fatigue are not independent phenomena but are interlocked through shared predictive dynamics, where errors in one domain propagate into the other. Fatigue can diminish agency by constraining the cognitive and energetic resources needed to support motor prediction, while disruptions in agency can exacerbate fatigue by undermining the accuracy of effort-cost forecasting. Thus, the relationship between agency and fatigue is bidirectional and recursive, sustained by the continuous evaluation of prediction errors.

An allostasis-based framework can therefore account for both the co-occurrence of agency disruptions and fatigue in clinical populations, as well as their everyday fluctuations in healthy individuals. Transient fatigue in healthy individuals reflects normal allostatic adjustments that reallocate energy to maintain system stability, whereas persistent pathological fatigue reflects the breakdown or chronic miscalibration of the predictive system governing these adjustments. By embedding agency and fatigue within this shared anticipatory framework, the allostatic model advances beyond the SAF model, offering a unified account of how the brain predicts, distributes, and evaluates energetic and cognitive demands in relation to action and control.

## 6 Discussion

This review aims to examine the sense of agency and the experience of fatigue through the lens of predictive processing. Taken separately, both phenomena have been explained in terms of how the brain anticipates, evaluates, and updates internal and external states. Examined together, however, a deeper organizing principle emerges: agency and fatigue are not just parallel constructs but perceptual consequences of the same predictive operations. Disturbances in these mechanisms can simultaneously undermine the feeling of control and amplify the experience of effort.

The central contribution of this review is to move beyond isolated accounts and propose an integrated framework in which agency and fatigue are two dynamically linked expressions of the same predictive system. The allostatic perspective provides the conceptual advance here. Whereas the Comparator Model of agency (Blakemore et al., 1998, 2001) and the Central Governor Model of fatigue (St Clair Gibson and Noakes, 2004) each formalize prediction within their own domain, the allostatic framework situates them within a common anticipatory system that influences both resources allocation and action ownership. This reframing has two key implications. First, it enables us to understand physiological fatigue in healthy individuals and pathological fatigue in clinical populations as points on the same predictive continuum, a concept that earlier models did not capture (Kuppuswamy, 2017, 2022). Second, it emphasizes the bidirectional nature of the relationship: fatigue can constrain the resources available for accurate action monitoring, while impaired agency can distort predictions of energetic and control costs, generating a self-reinforcing cycle of dysfunction.

This shift in perspective carries methodological and theoretical consequences. Current evidence remains fragmented, with behavioral studies often manipulating effort without assessing fatigue directly (Hon et al., 2013; Minohara et al., 2016), fatigue research rarely incorporating measures of agency (Di Vico et al., 2021; Kuppuswamy, 2022), and neuroimaging work tending to isolate interoceptive or motor networks rather than examining their interaction. These siloed approaches limit our ability to determine whether predictive disruptions truly span both domains. Progress will require integrative experimental designs that measure agency and fatigue within the same individuals, combining implicit and explicit agency markers, ecological effort assessments, and multimodal neuroimaging.

The implications of this integrated perspective extend well beyond theory. Fatigue is among the most common and disabling complaints across neurological and psychiatric conditions (Di Vico et al., 2021), and disturbances in agency likewise interfere with daily functioning and self-initiated action (Moccia et al., 2024). If both can be traced to predictive dysregulation within shared networks, then clinical assessment and treatment may benefit from moving away from symptom isolation. More broadly, an allostatic model encourages clinicians and researchers to treat agency disturbances and fatigue as mutually reinforcing symptoms that may require joint therapeutic strategies.

Despite its integrative value, the proposed allostasis-based model presents some limitations that should be acknowledged. First, the framework is primarily theoretical and rests on indirect evidence, as very few studies have simultaneously measured agency and fatigue within the same experimental design. Closely related to this issue, current neuroimaging findings pointing to partially overlapping networks remain predominantly correlational and do not provide causal evidence for a common mechanism linking agency and fatigue (Casamento-Moran et al., 2023; Kuppuswamy, 2022; Sperduti et al., 2011).

A second set of limitations concerns construct heterogeneity and temporal scale. Both agency and fatigue are multidimensional constructs: agency includes implicit and explicit components, whereas fatigue spans state and trait forms as well as mental and physical domains. The proposed allostatic model assumes a predictive coupling between the subjective experience of fatigue and the pre-reflective sense of agency, yet such coupling may not operate uniformly across the different dimensions of these constructs. Most experimental work to date has focused on transient, task-induced (state) fatigue, potentially overlooking the role of trait fatigue as a stable dispositional factor. It therefore remains unclear whether alterations in agency might be related to transient fatigue states, more stable trait-like predictive dispositions, or their interaction.

These limitations, however, should not be viewed as weaknesses, but rather as clarifying the current scope of the framework and pointing to concrete, testable predictions. Future research should therefore adopt integrative paradigms that assess agency and fatigue simultaneously, while explicitly distinguishing between different fatigue dimensions (e.g., state vs. trait).

A suitable approach would be a within-subject design combining implicit and explicit measures of agency (e.g., intentional binding, sensory attenuation, and agency judgments) with repeated assessments of subjective state fatigue across task progression, alongside validated measures of trait fatigue. According to our allostasis-based predictive model, both dimensions of fatigue are expected to influence agency, albeit at different temporal levels. Transient increases in state fatigue may dynamically modulate agency performance and judgments during task execution, reflecting a temporary reduction in perceived available resources. In contrast, trait fatigue may reflect a more enduring recalibration of predictive estimates about energetic and control demands. Consequently, individuals with higher trait fatigue may exhibit systematically altered agency signals already at baseline and show a different sensitivity to task-induced fatigue.

Considering state, trait, and pathological fatigue would allow researchers to disentangle transient fluctuations in internal state from more stable dispositional and clinical forms of fatigue, and to test whether these different dimensions differentially shape agency indices.

In conclusion, the evidence reviewed here suggests that agency and fatigue are best understood as interconnected outcomes of predictive regulation. By advancing an allostatic perspective, we offer a conceptual bridge between these phenomena and outline a unified anticipatory system that governs both perceived control and perceived effort.

Future research will be essential to test this account empirically. In particular, integrative paradigms that assess agency and fatigue simultaneously, computational models capturing prediction errors across domains, and longitudinal studies in clinical populations may help clarify the mechanisms linking these phenomena. Another promising direction concerns the potential influence of pharmacological agents that alter fatigue perception. Substances such as stimulants or other medications that modulate perceived effort and energy availability may also affect the relationship between fatigue and the sense of agency. Examining whether pharmacological modulation of fatigue alters agency signals could therefore provide an additional empirical test of the proposed framework. Such work has the potential not only to clarify the mechanisms linking agency and fatigue but also to inform more holistic diagnostic and therapeutic strategies.

## Data Availability

The original contributions presented in the study are included in the article/supplementary material, further inquiries can be directed to the corresponding authors.
